# Osteomyelitis caused by *Streptococcus intermedius* in immunocompetent adults — a case report and systematic literature review

**DOI:** 10.1007/s10096-023-04640-7

**Published:** 2023-07-20

**Authors:** Elyne M. Brunink, Lotje A. Hoogervorst, Koen Steentjes, Demien Broekhuis, Mark G. J. de Boer

**Affiliations:** 1 Department of Orthopaedics, Leiden University Medical Center, Leiden, 2333ZA the Netherlands; 2 Department of Biomedical Data Sciences & Medical Decision Making, Leiden University Medical Center, Albinusdreef 2, Leiden, 2333ZA the Netherlands; 3 Department of Infectious Diseases, Leiden University Medical Center, Leiden, 2333ZA the Netherlands; 4 Department of Clinical Epidemiology, Leiden University Medical Center, Leiden, 2333ZA the Netherlands

**Keywords:** *Streptococcus intermedius*, Osteomyelitis, Hematogenous, Immunocompetent, Infectious diseases

## Abstract

Hematogenous osteomyelitis caused by *Streptococcus intermedius* is rare, particularly in immunocompetent adults. The aim of this paper is to provide an overview of the clinical presentation, prognosis as well as treatment of this disease, with the focus on immunocompetent adults. Six medical literature libraries were searched to identify studies reporting on *Streptococcus intermedius* induced hematogenous osteomyelitis in immunocompetent adults. In addition, we presented a case of a 44-year-old man from our institution that is illustrative for this disease. Four case reports describing four patients were identified by this systematic literature review. Hence, the data of five patients (including our case) were assessed. The most common presenting symptom was localised pain, followed by fever. Portal entries were found in two patients (general periodontitis and necrotic dentition). The localisations of osteomyelitis were diverse: femoral (two patients), lumbar spine (two patients), and the iliac bone (one patient). Treatment strategies varied strongly, but antibiotics (penicillins) were administered in each case, and two patients underwent surgical debridement. Follow-up ranged from 2 weeks to more than 6 months; one patient died from septic shock. Only a very limited number of immunocompetent adults with *Streptococcus intermedius* induced hematogenous osteomyelitis have been described. Based on the available data, we summarised the clinical presentation, prognosis as well as treatment of hematogenous osteomyelitis caused by *Streptococcus intermedius* in this patient population.

## Introduction

*Streptococcus intermedius* is a beta-hemolytic Gram-positive bacteria that belongs to the *Streptococcus anginosus* group, commonly known as the *Streptococcus milleri* group, that further consists of *Streptococcus anginosus* and *Streptococcus constellatus* [1–4]. *Streptococcus intermedius* normally colonises in the mucosal sites of the oral cavity, upper respiratory tract, gastrointestinal tract, and the genitourinary tract [2, 5].

Infections caused by *Streptococcus intermedius* are often located in the head/neck region, such as craniofacial osteomyelitis and brain abscesses, with most infections occurring following dental and/or mandibular procedures [6–8]*.* Cases of hematogenous osteomyelitis of the long bones and the skeletal spine caused by *Streptococcus intermedius* have been rarely reported, particularly not in immunocompetent adults [9–14].

In this paper, we describe a case of *Streptococcus intermedius* caused femoral osteomyelitis in an immunocompetent 44-year-old male, and we review the current available literature with regard to *Streptococcus intermedius* induced hematogenous osteomyelitis in immunocompetent adults.

## Case report

**Fig. 1:**
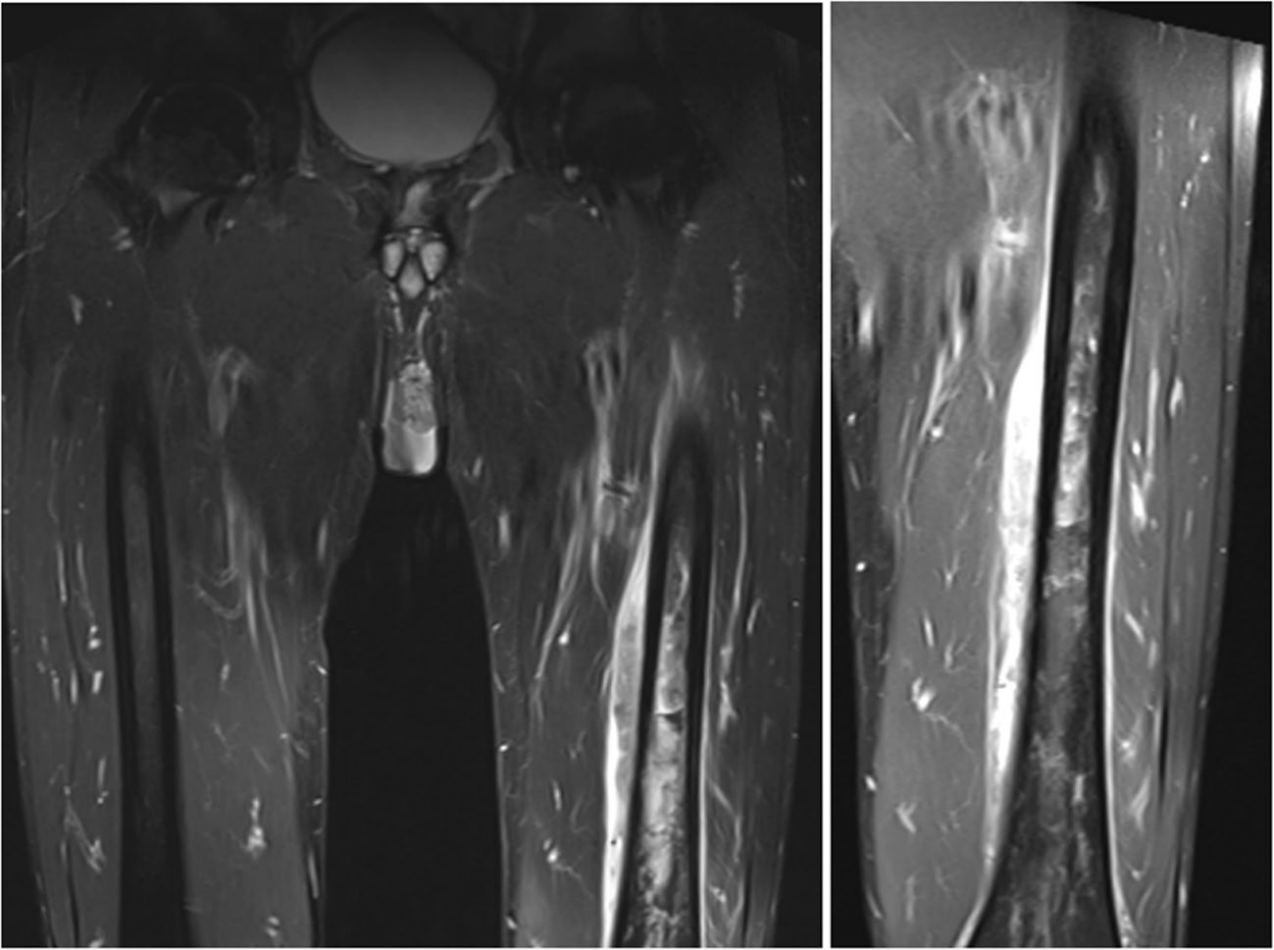
MRI scan (number 1) of both femurs (left) and a close-up of the left femur (right) showing a periosteal lesion in the left musculus vastus intermedius and a permeative process of the left femoral diaphysis

A 44-year-old man with no past medical history visited his general practitioner because of a 2-week history of a painful and swollen left upper leg and a sudden onset of fever (38.6 °C). There was no history of trauma. During physical examination, his left upper leg was painful and was slightly swollen. No skin abnormalities were observed, and a complete neurological examination also revealed no abnormalities. Blood test results revealed elevated inflammatory markers: CRP 112 mg/L (normal range: < 5.0 mg/L) and leucocytes 13.0 10*9/L (normal range: 4.0–10.0 10*9/L). An MRI (number 1, Fig. 1) showed a periosteal soft tissue lesion of the musculus vastus intermedius measuring 28 × 11 × 140 mm and a permeative process of the femoral diaphysis with a long intramedullary extending abnormal T2 signal, and a plain radiograph of the left femur (not shown) showed a small periosteal lesion of the posterior femoral diaphysis. Due to the radiological suspicion of malignancy, the patient was referred to our institution, specialised in the management of bone cancer.

At the time of first presentation, physical examination of the left upper leg revealed no visible signs of infection, and the leg had full range of motion. The left upper leg was however severely painful both during walking and in rest (numeric rating scale of 8 out of 10, where 0 is no pain and 10 is the worst pain imaginable). Vital signs were within normal range. Due to the severe pain (under oral analgesics), the patient was admitted to the orthopaedic ward for analgetic management and diagnostic work-up.

**Fig. 2:**
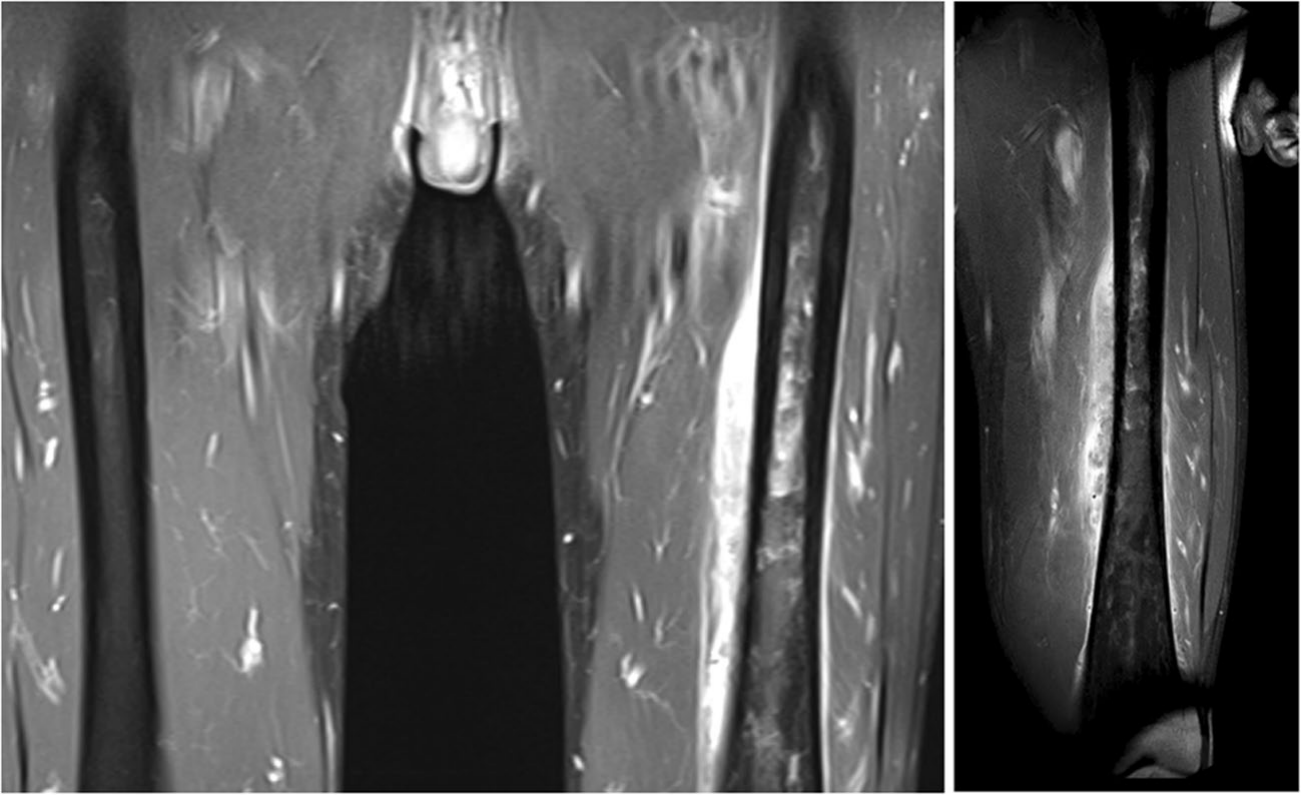
MRI scan (number 2) of both femurs (left) and a close-up of the left femur (right) showing a parosteal lesion, oedema, and enhancement of the musculus vastus intermedius (28 × 11 × 140 mm) including general oedema in the surrounding musculature and permeative process of the left femoral diaphysis

Routine blood test results at the time of admission again shared elevated inflammatory markers, and a second MRI (Fig. 2) with gadolinium contrast showed the process to be rapid enhancing with diffusion restriction and general oedema in the surrounding musculature. The differential diagnosis consisted of a small cell tumour and osteomyelitis. To obtain tissue samples to distinguish between the aforementioned diagnosis, a Jamshidi bone biopsy was performed. Tissue samples were analysed for bacterial- and fungal-pathogens (Gram-stain and culture) as well as histological assessment.

Initial bacterial culture test showed the bacteria *Streptococcus intermedius*, confirming the diagnosis of osteomyelitis. Penicillin (6 million units/24 h intravenous) together with metronidazole (three times daily, 500 mg orally) were started after susceptibility tests showing penicillin and clindamycin sensitivity. Due to the identified pathogen (which normally colonises in the mucosal sites of the oral cavity, upper respiratory tract, gastrointestinal tract, and genitourinary tract [2, 5]) and suspected hematogenous dissemination, a dental examination, CT-scan of the brain, and an ultrasound of the abdomen were performed which all showed no abnormalities.

**Fig. 3:**
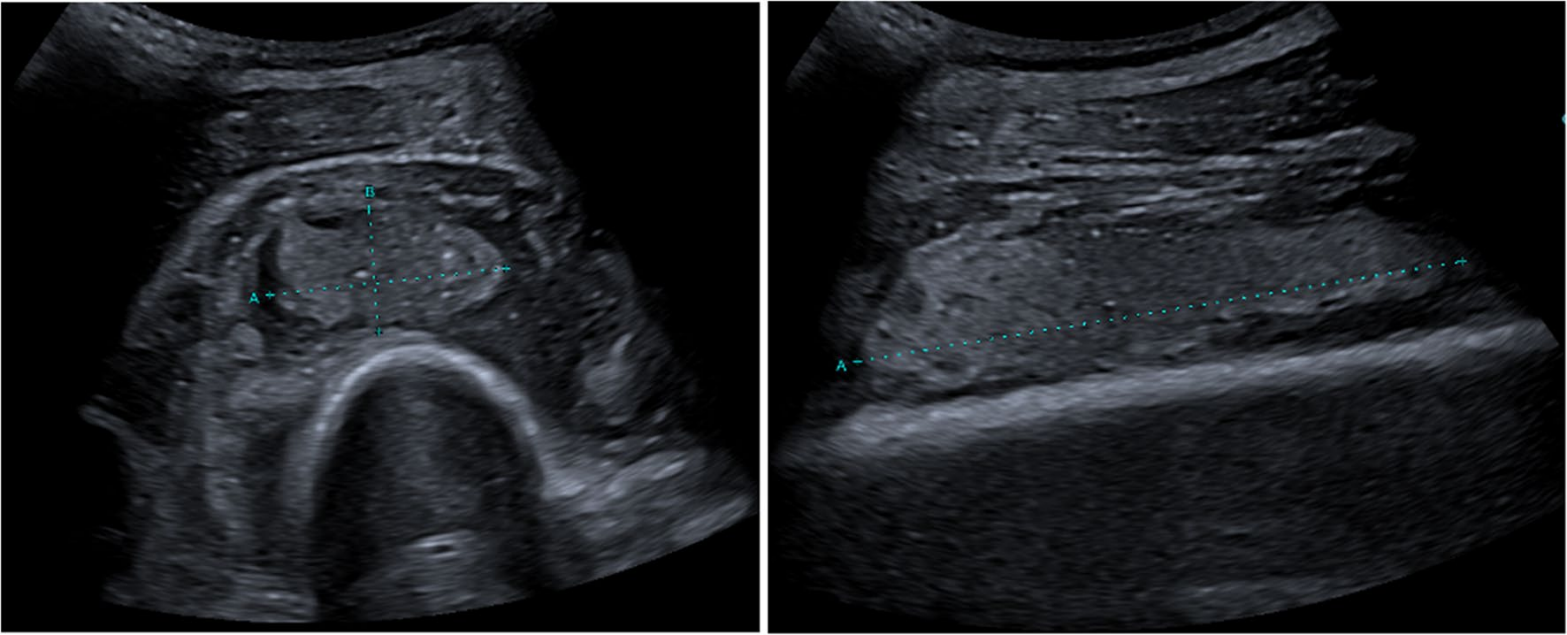
Ultrasound of the left femur showing abscesses near the musculus vastus lateralis (8.0 × 3.3 × 1.7 cm) and musculus intermedius (6.0 × 2.7 × 1.3 cm)

In the following days, the patient experienced increasing pain in his leg. An ultrasound of the left upper leg showed two new abscesses: one near the musculus vastus lateralis (8.0 × 3.3 × 1.7 cm) and one near the musculus intermedius (6.0 × 2.7 × 1.3 cm) (Fig. 3). Consequently, two ultrasound-guided needle aspirations of the abscesses were conducted aiming to relieve pain and to prevent further progression of the abscesses. Two days later, persistent abscess formation was confirmed by ultrasound, and given that at this stage the histopathology results showed no malignant cells, surgical local debridement including intramedullary reaming of the femoral shaft was performed. The same regime of antibiotics (penicillin and metronidazole) was continued post-operatively.

The combination of the surgical management and antibiotics resulted in clinical improvement with no clinical signs of infection and inflammatory markers were within normal range approximately 1 month post-operatively. The patient was able to ambulate with two crutches and was discharged out of hospital 7 days post-operatively, with the continuation of antibiotics. Two weeks after the start of penicillin, the penicillin was switched to amoxicillin 1000 mg orally four times daily for a total duration of 6 weeks post-operatively. In the absence of anaerobic microorganisms, metronidazole was stopped after 11 days of treatment. The patient was monitored at the orthopaedic outpatient clinic once every 2 weeks for the duration of 2 months, followed by once every month until 6 months post-operatively. During his follow-up period, no complications or re-infection occurred, and the patient showed full recovery.

## Literature review

The review was conducted according to the Preferred Reporting Items for Systematic Reviews and Meta-Analyses (PRISMA) 2020 guidelines [15]. Six medical literature libraries (Embase, Emcare, Cochrane Library, Medline, PubMed, and Web of Science) were searched for publications using a systematic search created by EB together with an experienced medical librarian (Appendix 1). The list of references was exported to EndNote (Version X9, Clarivate Analytics, Philadelphia, USA) to remove duplicate articles and subsequently exported to the web application Rayyan (Doha, Qatar) for study selection.

Titles and abstracts were independently screened by two reviewers (EB and LH). Inclusion criteria were clinical studies reporting on (i) hematogenous osteomyelitis caused by *Streptococcus intermedius*; (ii) immunocompetent patients; (iii) adults (≥ 18 years old); (iv) osteomyelitis located in the extremities and in the sacral, lumbar, or thoracic spine. Exclusion criteria were (a) osteomyelitis located in the head/neck region; (b) diabetic foot osteomyelitis; (c) language other than Dutch, English, or German; and (d) (peri)prosthetic joint infections. References of included studies were checked for eligible studies.

Data was extracted independently by EB and LH using a prespecified format and was presented using descriptive statistics.

A total of 298 publications were identified using the systematic search. After the removal of duplicates, 250 publications were screened for eligibility based on title and abstract. Thereafter, 43 full texts were assessed of which four studies fulfilled the inclusion criteria **(**Appendix 2) describing a total of four immunocompetent adults with *Streptococcus intermedius* induced hematogenous osteomyelitis [9, 11, 13, 14]. These cases, as well as the patient in the present paper, are summarised in Table 1.

**Table 1:** Clinical characteristics and outcomes of immunocompetent adults with hematogenous osteomyelitis caused by *Streptococcus intermedius*

	Calza (2001)[9]	Schattner (2014)[14]	Janssen (2017)[11]	Quast (2017)[13]	Current paper
Age (years)	30	55	57	63	44
Sex	Male	Male	Male	Male	Male
Initial clinical presentation + duration of symptoms before first presentation	- Asthenia, chills, intermitted fever, lack of appetite, localised pain - 2 weeks	- Anorexia, growing lassitude, localised pain, night sweats, weight loss - 2 weeks	- Localised pain - Not reported	- Arthralgia, fever, localised pain, rigor - 4 months	- Fever, localised pain, localised swelling - 2 weeks
Location of osteomyelitis	Ilium bone	Lumbar spine	Femoral bone	Lumbar spine	Femoral bone
Inflammatory markers	Leucocytosis (16,800 cells/ul), neutrophilia (14,450 cells/ul)	Elevated CRP (191 mg/L), leucocytosis (26.4 × 10*3/uL)	Elevated CRP (110 mg/l), leucocytosis (9.9 × 10*9/l)	Leucocytosis (31,700 × 10*9/l)	Elevated CRP (173.8 mg/L), leucocytosis (14.04 × 10*9/L)
Diagnostic tools and findings —focusing on the location of osteomyelitis	- CT scan (pelvic): osteolytic lesion and abscess	- CT scan (whole body): osteomyelitis - MRI scan: discitis, osteomyelitis, abscess	- MRI scan (femur): lytic lesion, fluid collection - Radiograph (femur): lytic lesion and pathological fracture	- MRI scan (1) (lumbar spine): fluid collection, discitis, oedema, osteomyelitis - MRI scan (2) (lumbar spine): discitis, abscess	- MRI scan (1 and 2) (femur): periosteal lesion, permeative process - Radiograph (femur): periosteal lesion - Ultrasound (1 and 2) (femur): abscesses
Diagnostic tools and findings —identifying portal entry	Electocardiogram, radiograph (chest and oral cavity), urinalysis, ultrasound (abdomen)	Colonoscopy, echocardiography, electrocardiography, radiograph (chest), urinalysis	Bone scintigraphy, CT scan (abdomen and chest), echocardiography (transthoracic and transesophageal)	Echocardiogram (transesophageal), radiograph (chest and oral cavity), urinalysis	CT scan (brain), ultrasound (abdomen)
Portal of entry	Periodontitis	Not reported	Not reported	Necrotic dentition	None
Treatment	Antibiotics	Antibiotics and surgery	Antibiotics and surgery	Antibiotics	Antibiotics and surgery
Duration of follow-up	9 weeks	Not reported	Not reported	Not reported	6 months
Clinical outcome	Complete resolution of bone lesions	“Almost full recovery”	Death due to septic shock including multiple organ failure	Not reported	Full recovery

## Discussion

### Patients’ characteristics, clinical presentation, and diagnostic assessment

All patients were male, with a median (range) of 55 (30–63) years. All patients, including ours, presented with localised pain. Other described presenting complaints were anorexia, asthenia, fever, generalised arthralgia, growing lassitude, night sweats, rigor, and weight loss. Time from symptoms to initial hospital presentation ranged from 2 weeks to 4 months.

As *Streptococcus intermedius* normally colonises in the mucosal sites of the oral cavity, upper respiratory tract, gastrointestinal tract, and genitourinary tract [2, 5], it is important to consider additional diagnostic interventions such as echocardiography and urinalysis to assess for portal entries and thereby treating underlying conditions. Portal of entry was found in two patients which included general periodontitis and necrotic dentition.

The localisations of osteomyelitis were diverse: femoral (two patients), lumbar spine (two patients), and the iliac bone (one patient). MRI was the most frequently used diagnostic tool and mostly showing bone alterations with surrounding abscess(es) and/or fluid collection(s).

### Treatment and patient outcomes

Treatment strategies varied strongly, but antibiotics (penicillin) were administered in each case. As *Streptococcus intermedius* is able to express the hydrolytic enzymes which are responsible for pus formation, abscesses are often seen in *Streptococcus intermedius* induced infections [2]. To limit and/or relieve abscess formation, surgical debridement was performed in two cases (including ours). In addition, as *Streptococcus intermedius* has been commonly associated with recurrent abscesses [1, 16, 17], it was noticed that multiple surgical debridements were often required. Follow-up ranged from 2 weeks to more than 6 months. One patient died from septic shock.

### Study limitations

Some limitations of the current study need to be addressed. First, the number of studies in the current literature that report on *Streptococcus intermedius* induced hematogenous osteomyelitis in immunocompetent is low. As shown in the flowchart of the study selection and inclusion process (Appendix 2), most studies had to be excluded as the osteomyelitis was caused by another aetiological agent than *Streptococcus intermedius.* We decided to only include studies focusing on *Streptococcus intermedius* instead of the entire *Streptococcus milleri* group (including *Streptococcus anginosus*, *Streptococcus constellatus*, and *Streptococcus intermedius)* as previous literature has shown that virulence factors as well as clinical syndromes may differ between these three aetiological agents [18]. Older case reports in which the causing agent was defined as, e.g. *Streptococcus milleri* actually could or might have been included if modern microbiologic methods and taxonomy would have been applied. Secondly, publication bias (e.g. underreporting cases with bad clinical outcomes such as mortality) is a well-known problem of systematic reviews and could have influenced our results. Last, we decided to focus only on adults. To the best of our knowledge, only two cases of *Streptococcus intermedius* induced hematogenous osteomyelitis in immunocompetent children (< 18 years old) have been described before including a case of sacroiliitis [19] and a case of right femoral osteomyelitis [20].

## Conclusions

Hematogenous osteomyelitis caused by *Streptococcus intermedius* is rare, particularly in immunocompetent adults. It may lead to severe destruction of affected bones, including abscess forming which might lead to longstanding or chronic complaints and even to mortality. To prevent this, adequate medical treatment is required and, in most cases, also additional surgical interventions. Unfortunately, the available evidence is insufficient to definitively determine the most effective treatment. Based on our experience, we recommend treating *Streptococcus intermedius* induced osteomyelitis with intravenous antibiotics in combination with surgical debridement. Additional diagnostic assessment is indicated to assess for other foci of infection.

## Figures and Tables

**Fig. 4:**
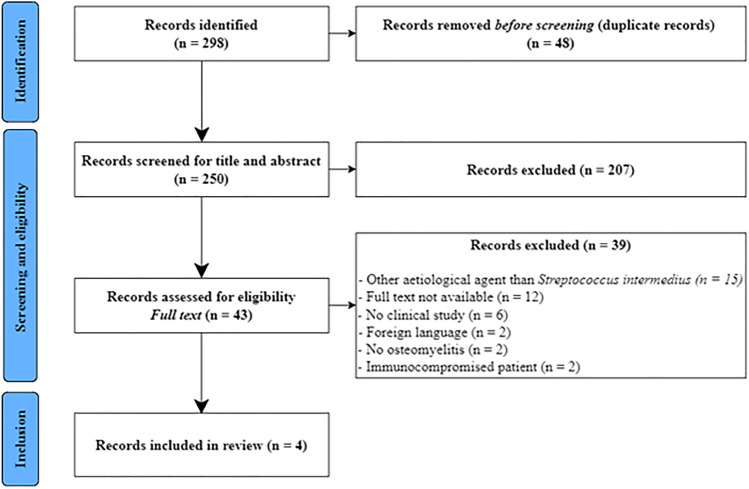
Flowchart of study selection and inclusion process

## Data Availability

The data used to support the findings of this study are included within the article.

## Appendix 1. Systematic search

((“osteomyelitis”[MeSH Major Topic] OR “osteomyelitis” [Text Word] OR “sacroiliitis” [Text Word] OR “osteitis” [Text word]) AND (“Streptococcus milleri” [MeSH Major Topic] OR “streptococcus milleri group” [MeSH major topic] OR “streptococcus intermedius” [MeSH major Topic] OR “Streptococcus anginosus group” [MeSH Major Topic] OR “Streptococcus anginosus” [Mesh Major Topic] OR “streptococcus intermedius” [Text Word] OR “Streptococcus anginosus group” [Text word] OR “Streptococcus anginosus” [Text Word] OR “Streptococcus milleri group” [Text word] OR “streptococcus milleri” [Text Word])).

## Appendix 2

Please see Fig. 4.
